# Isolated Vertigo as an Early Sign of Anti-GQ1b-Positive Miller Fisher Syndrome: Expanding the Spectrum

**DOI:** 10.7759/cureus.98995

**Published:** 2025-12-11

**Authors:** Noor Widyani Ahmad Shahaime, Mazaya Mahmud, Othmaliza Othman, Wan Haslina Wan Abdul Halim

**Affiliations:** 1 Department of Ophthalmology, Hospital Canselor Tuanku Muhriz, Universiti Kebangsaan Malaysia (UKM), Kuala Lumpur, MYS; 2 Department of Ophthalmology, Universiti Putra Malaysia (UPM), Kuala Lumpur, MYS; 3 Faculty of Medicine and Health Sciences, University College Sedaya International (UCSI University), Kuala Lumpur, MYS

**Keywords:** acute vestibular syndrome, anti-gq1b antibody, ataxia, guillain-barre spectrum, immune-mediated neuropathy, miller fisher syndrome, neurology, neuro-ophthalmology, ophthalmoplegia, vertigo

## Abstract

Miller Fisher syndrome (MFS) occupies a distinct corner of Guillain-Barré syndrome (GBS), typically presenting with a triad comprising ophthalmoplegia, ataxia, and areflexia. While diplopia and limb incoordination are typically early features, isolated vertigo as a prodromal symptom is exceedingly uncommon and often misdiagnosed as benign vestibular disease. We report a 56-year-old Malay man who presented twice to the emergency department with acute, continuous vertigo and was initially treated symptomatically before subsequently developing bilateral ophthalmoplegia and ataxia. Anti-ganglioside (anti-GQ1b) antibody testing was positive, confirming the diagnosis of MFS, and electrophysiological studies revealed early GBS-spectrum changes. The patient responded well to intravenous immunoglobulin (IVIG) and made a full recovery within six weeks. This case illustrates that vestibular symptoms can occur early within the anti-GQ1b spectrum and may easily be overlooked. It also underscores the importance of careful, repeated neurological and ophthalmological examinations. Early recognition and treatment are critical to ensuring successful recovery.

## Introduction

Miller Fisher syndrome (MFS) represents a small but distinct subset within the Guillain-Barré syndrome (GBS) spectrum, a condition characterized by immune misrecognition. The condition was first described by Collier in 1932 and later defined by Fisher in 1956 as a complex comprising ophthalmoplegia, ataxia, and areflexia [1]. Although MFS is rare, the prevalence is higher in parts of Asia [2]. Such epidemiological differences are likely shaped by infectious exposures, underlying host susceptibility, and regional variation in diagnostic practice [3].

While the triad defines the syndrome, early manifestation may be subtle or incomplete. Isolated vertigo, in particular, can precede other neurological signs and lead to diagnostic uncertainty in acute care settings, where peripheral vestibular disorders are far more common. Recognizing this diagnostic pitfall is important, as vestibular involvement has increasingly been described across the expanding anti-GQ1b spectrum [4,5].

MFS typically follows an upper respiratory tract or gastrointestinal infection, and molecular mimicry between microbial antigens and neuronal ganglioside GQ1b triggers an autoimmune response that attacks the paranodal regions of ocular and vestibular nerves. This pathophysiological mechanism links the clinical involvement observed in MFS and its related anti-GQ1b spectrum [6,7].

We reported a rare case of anti-GQ1b-positive MFS that began with isolated vertigo, which later progressed to the full clinical triad. This case underscores the potential for early vestibular involvement within the anti-GQ1b spectrum and diagnostic pitfalls within the expanding anti-GQ1b spectrum.

## Case presentation

A 56-year-old Malay man with well-controlled asthma presented with persistent vertigo for two weeks. The vertigo was described as a continuous spinning sensation that worsened with movement. There were no accompanying symptoms, such as diplopia, facial asymmetry, slurred speech, limb weakness, numbness, tinnitus, or hearing impairment. He denied any recent upper respiratory tract infection, gastrointestinal illness, fever, trauma, smoking, drug or alcohol use, and was not on any chronic medication.

He visited the emergency department twice during the initial two-week period. On both visits, he was hemodynamically stable, his pupils were reactive, and no nystagmus was observed. His symptoms improved with prochlorperazine and were treated as peripheral vertigo.

**Figure 1:**
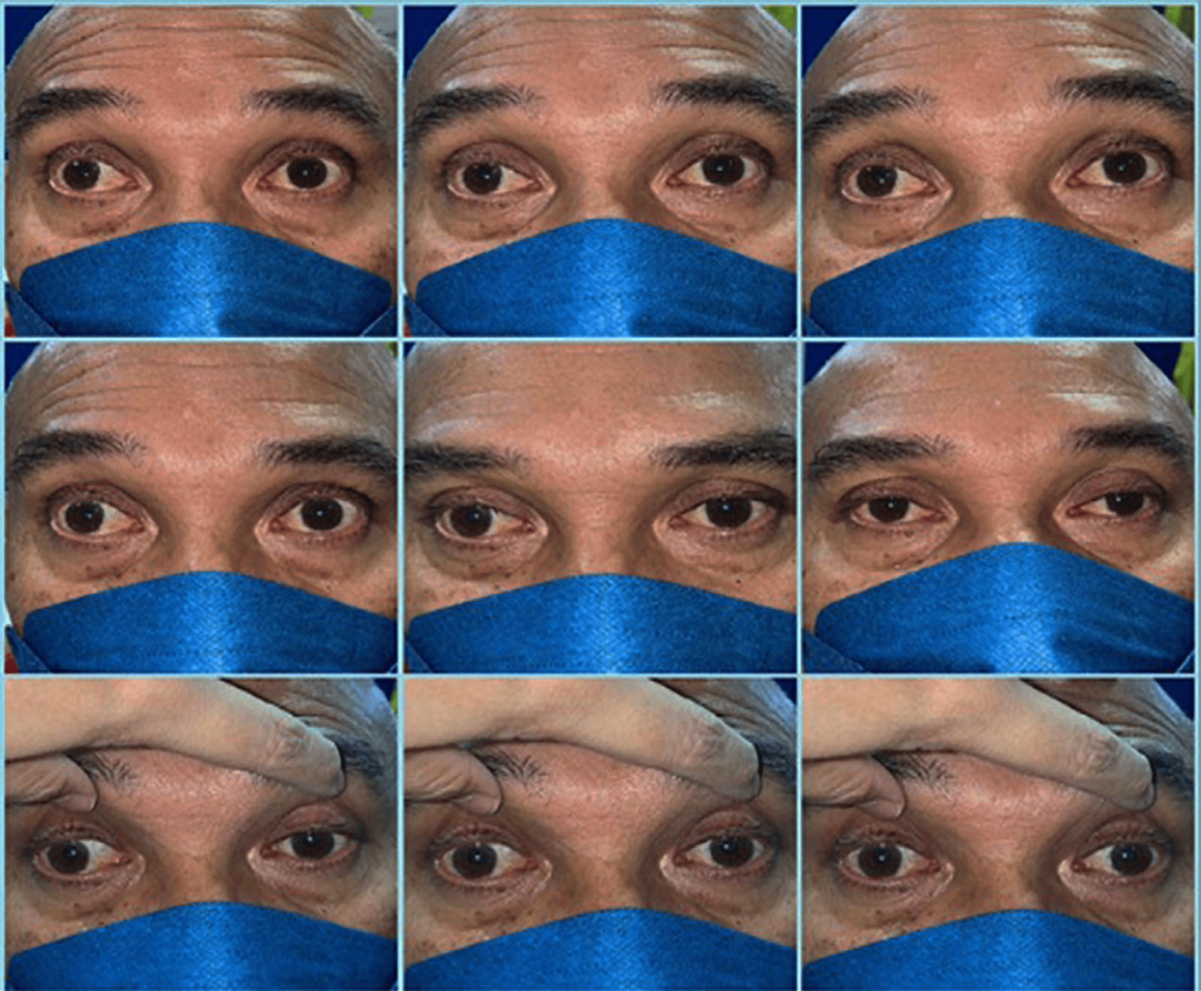
Complete ophthalmoplegia at presentation. The patient was instructed to attempt upgaze, downgaze, left gaze, and right gaze, with very limited to almost no extraocular movement observed in any direction.

Approximately one week after his second visit, he noticed increasing unsteadiness. He reported difficulty detecting objects in his peripheral visual field unless he turned his head, although he denied having diplopia or blurred vision. Examination revealed complete external ophthalmoplegia (Figure 1), generalized areflexia, and broad-based ataxia. Limb strength was intact, and there were no signs of facial weakness, sensory deficit, or bulbar involvement.

**Figure 2:**
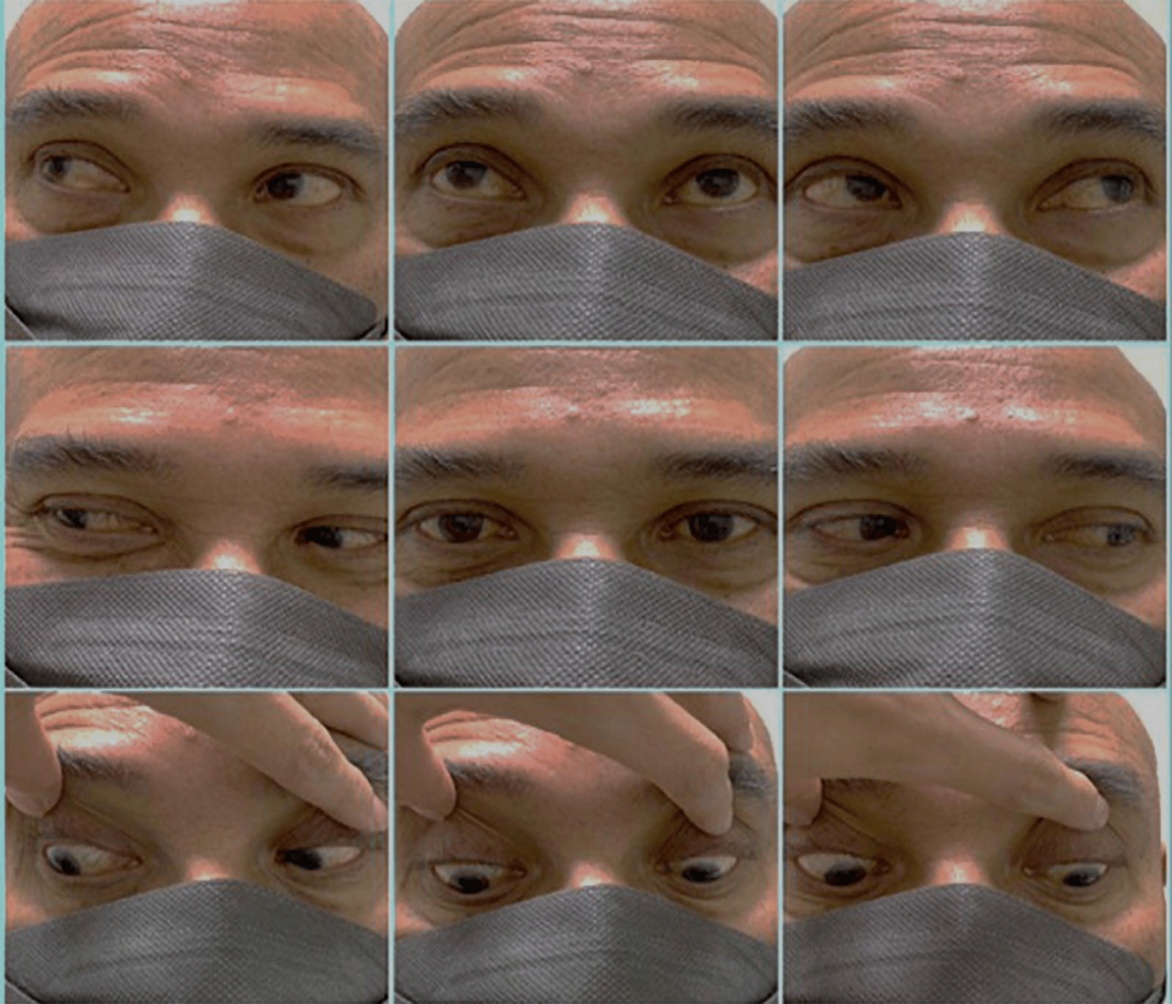
Resolution of ophthalmoplegia at six weeks. The patient demonstrated full extraocular movements in all gaze directions, consistent with complete recovery of ocular motility.

Initial laboratory investigations, including full blood count, renal profile, electrolytes, and glucose levels, were all within normal limits. Computed tomography of the brain (CT brain) showed no acute intracranial pathology. The patient was subsequently co-managed with the neurology team. Nerve conduction studies (NCS) showed normal distal motor and sensory conduction across the median, ulnar, peroneal, and sural nerves. However, H-reflexes were absent bilaterally, indicating early proximal demyelinating involvement, a common electrophysiological finding in the GBS spectrum. Serum anti-GQ1b antibody testing returned positive, confirming the diagnosis of MFS. He received IVIG (0.4g/kg/day) for five days. His reflexes improved during the admission, and his ophthalmoplegia (Figure 2) and ataxia were fully resolved by the six-week follow-up.

## Discussion

Mechanistic bridge: Molecular mimicry and vestibulo-ocular vulnerability

The pathophysiological basis of MFS is well established and centers on the formation of anti-GQ1b IgG antibodies through molecular mimicry. Campylobacter jejuni is a well-recognised precursor infection in GBS, and its lipo-oligosaccharides share structural similarity with ganglioside GQ1b, providing the immunological link that drives antibody-mediated neuropathy [2,6]. The expression of GQ1b is highest in the ocular motor, vestibular, and proprioceptive fibres, making them vulnerable to damage. Antibodies bind to these paranodal regions, interfere with axonal conduction, and result in a range of characteristic clinical features observed across the spectrum. These features include symptoms such as ophthalmoplegia, ataxia, and areflexia. If the vestibular fibers are affected, vertigo or oscillopsia may emerge before other neurological signs [6,7]. 

While a preceding infection is often noted in classical cases, our patient did not have any prior infectious trigger. This is consistent with reports describing a subset of anti-GQ1b-positive patients without a clear antecedent infection, which contributes to the diagnostic challenges in identifying the spectrum in early presentations [6,8].

Isolated vertigo and early presentations within the anti-GQ1b spectrum

Patients with classical MFS typically present with ophthalmoplegia in nearly all cases, ataxia in 94%, and areflexia in approximately 90%. These symptoms usually emerge within the first one to four days [8]. In contrast, conditions within the anti-GQ1b spectrum may develop sequentially or remain incomplete, exhibiting a non-classical order depending on the initial site of antibody binding [4].

Although isolated vertigo is rarely highlighted in classical MFS studies, early ocular motor signs, such as nystagmus, have been documented, suggesting early vestibulo-ocular involvement [8]. Supporting this, Jung et al. described that atypical MFS initially presented with dizziness, imbalance, and gait unsteadiness, underscoring that vestibular pathways may have been affected way earlier than traditionally recognized [9]. While specific distributions of GQ1b in vestibular pathways are still not fully understood, clinical observations have consistently demonstrated that vestibular symptoms can manifest before the onset of more apparent neuro-ophthalmic symptoms in certain anti-GQ1-associated conditions [8-11]. This evidence expands the understanding of early presentations within the anti-GQ1b spectrum.

Recent research has further refined our understanding by grouping MFS, vestibular-predominant forms, Acute Ophthalmoparesis (AO), Bickerstaff Brainstem Encephalitis (BBE), and the overlap of MFS and GBS into a single, dynamic anti-GQ1b continuum [4,5]. In this spectrum, isolated vertigo may represent the earliest clinical manifestation, possibly appearing even before the hallmark triad of symptoms. Evidence from acute vestibular syndrome (AVS) studies further supports this notion, as patients who initially present with acute vertigo or dizziness often later develop ocular motor abnormalities [10]. Our patient followed this trajectory closely, experiencing two weeks of isolated vertigo before progressing to complete MFS. A similar vertigo-first onset has been described only once in a dedicated case report [11].

Recognizing persistent or unexplained vertigo as a possible early presentation within the anti-GQ1b spectrum reframes diagnostic expectations. This approach justifies repeated and comprehensive neurological and ophthalmic assessment and supports early anti-GQ1b testing when symptoms are evolving.

Differential diagnosis and diagnostic pitfalls

Vertigo as the initial and isolated symptom can mimic a wide range of conditions, particularly when ocular signs or areflexia have yet to emerge [12]. This was evident in our case, where the initial presentation closely resembled that of peripheral vestibular disease, which delayed consideration of MFS. The limited recognition of vertigo in MFS likely stems from the rare occurrence, as well as the common diagnostic approach used in acute care, where isolated vertigo is often attributed to peripheral vestibulopathies or posterior circulation strokes [13,14]. A structured evaluation is therefore essential to exclude other potential causes of emergency, before attributing symptoms to immune-mediated disorders. Peripheral vestibulopathies, such as benign paroxysmal positional vertigo (BPPV) or vestibular neuritis, are common, especially in older adults. Central causes, such as posterior circulation strokes or mass lesions, may present without apparent focal deficits and must be excluded first [13-15]. The HINTS (Head Impulse, Nystagmus, and Test of Skew) examination has been demonstrated to be more effective than early MRI in differentiating central and peripheral vertigo [13]. Moreover, neuromuscular and metabolic conditions such as myasthenia gravis, electrolyte imbalances, endocrine disturbances, and drug toxicity may also create overlapping neurology and ophthalmic features [12,15]. Table 1 summarizes the major differential diagnoses and their distinguishing features relevant in the acute setting.

**Table 1:** Differentiating Miller Fisher syndrome (MFS) and other causes of acute vertigo. This table summarizes the principal differential diagnoses and distinguishing clinical features of MFS compared with other causes of acute vertigo. Data summarized from key literature sources as follows: MFS [4,8-12,15], posterior circulation stroke [12-15], space-occupying lesion [14,15], cardiac arrhythmia [14,15], benign paroxysmal positional vertigo/vestibular neuritis [13-15]; electrolyte imbalances/endocrine disturbance or drug toxicity [14,15]; myasthenia gravis [12]. NCS, nerve conduction study; IVIG, intravenous immunoglobulin; HINTS, head impulse, nystagmus, and test of skew; MRI, magnetic resonance imaging; DWI, diffusion-weighted imaging; ECG, electrocardiogram; BPPV, benign paroxysmal positional vertigo; vHIT, video head-impulse test; RNS, repetitive nerve stimulation; AChR, acetylcholine receptor; MuSK antibodies, muscle-specific kinase antibodies

Condition	Typical presentation	Key ocular/neurological features	Investigations/ancillary tests	Red flags/distinguishing features
Miller Fisher Syndrome	Ophthalmoplegia, ataxia, areflexia, may begin with vertigo	Areflexia with preserved limb power, ophthalmoplegia	NCS (may show sensory-predominant changes), anti-GQ1b serology	Progression beyond the isolated vertigo, evolving new neuro-ophthalmic features
Posterior Circulation Stroke	Continuous vertigo, vomiting, gait instability	Direction-changing gaze-evoked nystagmus	HINTS, MRI brain with DWI, vascular work-up	Sudden onset, notable vascular risk factors, abnormal HINTS
Space Occupying Lesion (Posterior Fossa Tumor bleeding/Herniation)	Sudden vertigo, vomiting, altered mental consciousness	Progressive focal cranial nerve deficits, variable ocular motor palsies/skew	HINTS, MRI brain/orbit with contrast	Progressive symptoms, features of raised intracranial pressure (e.g., headache, vomiting, papilledema)
Cardiac Arrhythmia	Episodic or persistent/presyncope dizziness (perceived as vertigo) ± palpitation	No nystagmus/neurological deficits	ECG, cardiac enzyme	Syncope, exertional episodes, irregular rhythm
BPPV/Vestibular Neuritis	Episodic positional (BPPV) or prolonged vertigo (neuritis)	No ophthalmoplegia or areflexia, normal limb strength	Dix-Hallpike (BPPV), caloric testing, vHIT	Triggered by movement of the head (BPPV), normal pupils, no diplopia, no limb weakness
Electrolyte Imbalance/Endocrine Disturbances/Drug Toxicity	Dizziness, oscillopsia, confusion, tremor	Non-specific	Glucose, electrolytes, thyroid panel, Selective drug levels	Linked to medication or metabolic derangement, improves with correction or drug withdrawal
Myasthenia Gravis	Fluctuating ptosis/diplopia without ataxia or areflexia	Fatigability test positive, intact reflexes	Ice-pack test, RNS (signal decrement), AChR/MuSK antibodies	Worsening at evening or repetitive movement, reflexes preserved

In patients with persistent or unexplained vertigo, it is crucial to perform repeated neuro-ophthalmic examinations, as signs associated with the anti-GQ1b spectrum may evolve over time [4,9,10]. This approach increases the likelihood of identifying subtle signs that may appear before the classical triad.

Treatment and outcomes

Corticosteroids have shown no therapeutic benefit in GBS [16], and similarly, are not recommended for MFS given the shared immunopathological mechanism and guideline extrapolations [17]. First-line therapy for MFS remains IVIG or plasma exchange (PLEX), both of which exhibit comparable efficacy. IVIG is generally favored for its practicality in administration and overall tolerance. Spontaneous recovery can happen; however, early treatment is recommended when neurological deficits are functionally significant, progressive, or when diagnostic delay is expected, particularly in vestibular-predominant cases [16,17]. Emerging therapeutic approaches such as FcRn blockade, enzymatic IgG reduction, and complement inhibition are currently being explored for patients with severe or refractory disease [17].

Our patient achieved complete recovery by six weeks of IVIG, mirroring the excellent prognosis described in classical MFS [8], reinforcing the value of early recognition, timely referral, and prompt initiation of immunotherapy [16,17].

## Conclusions

Persistent vertigo without peripheral vestibular features may, in rare instances, illustrate an early presentation of anti-GQ1b-positive MFS. This case highlights the need for comprehensive and repeated ophthalmology, neurology, and vestibular assessments when vertigo remains unexplained or evolves. Early anti-GQ1b testing can facilitate a timely diagnosis. Hence, recognizing these diverse presentations within the broader anti-GQ1b continuum enables prompt immunotherapy and favorable neurological recovery.
